# Matrix Effects on the Delivery Efficacy of Bifidobacterium animalis subsp. *lactis* BB-12 on Fecal Microbiota, Gut Transit Time, and Short-Chain Fatty Acids in Healthy Young Adults

**DOI:** 10.1128/mSphere.00084-21

**Published:** 2021-07-07

**Authors:** Zhaoyong Ba, Yujin Lee, Huicui Meng, Penny M. Kris-Etherton, Connie J. Rogers, Zachery T. Lewis, David A. Mills, Emily J. Furumoto, M. Laura Rolon, Jennifer A. Fleming, Robert F. Roberts

**Affiliations:** a Department of Food Science, The Pennsylvania State University, University Park, Pennsylvania, USA; b Department of Nutritional Sciences, The Pennsylvania State University, University Park, Pennsylvania, USA; c Department of Food Science and Technology, University of California, Davis, California, USA; Baylor College of Medicine

**Keywords:** probiotics, BB-12, gut microbiota, short-chain fatty acids, gut transit time

## Abstract

Probiotics are consumed in fermented dairy products or as capsules for their putative health benefits. However, little research has been done to evaluate the effects of the delivery matrix on the health benefits of probiotics in humans. To examine the effects of delivering Bifidobacterium animalis subsp. *lactis* BB-12 (BB-12) (log_10_ 10 ± 0.5 CFU/day) via a yogurt smoothie versus a capsule, we monitored the fecal microbiota, gut transit times (GTTs), and fecal excretion of short-chain fatty acids (SCFAs) in healthy adults. In a randomized, four-period, crossover study performed in a partially blind manner, 36 adults were recruited and randomly assigned to four treatments: control yogurt smoothie (YS), yogurt smoothie with BB-12 added prefermentation (PRE), yogurt smoothie with BB-12 added postfermentation (POST), and capsule containing BB-12 (CAP). Participants’ fecal microbiota was assessed using 16S rRNA sequencing, GTTs via SmartPill, and fecal SCFAs by gas chromatography (GC) before (baseline) and after each intervention. Participants had significantly higher percentage of Streptococcus after consuming YS versus CAP (*P* = 0.01). *Bifidobacterium*-specific terminal restriction fragment length polymorphism analysis revealed a significantly higher percentage of B. animalis after consuming PRE and POST compared to baseline, YS, CAP, and final washout (*P* < 0.0001). The predominant SCFAs were negatively correlated with GTTs. Consumption of BB-12 delivered in a yogurt smoothie or capsule did not significantly alter the composition of the gut microbiota, GTTs, or fecal SCFA concentration of the study cohort. However, daily consumption of BB-12 in yogurt smoothie may result in higher relative abundance of *B. animalis* in healthy adults. (This trial has been registered at ClinicalTrials.gov under identifier NCT01399996.)

**IMPORTANCE**Bifidobacterium animalis subsp. *lactis* BB-12 is a probiotic strain that has been used worldwide since 1985. It has commonly been delivered in fermented dairy products for perceived benefits associated with gut health and enhanced immune function. In addition to fermented dairy products, many new probiotic-containing alternatives such as probiotic-containing juice, probiotic-containing chocolate, and capsules have been developed. While these products provide more options for people to access probiotics, little research has been done on the effect of delivery matrix (dairy versus nondairy) on their efficacy in humans. In addition, it was unclear how yogurt fermentation may influence the survival of BB-12 in the product or on its performance *in vivo.* The significance of our study is in simultaneously assessing the effect of BB-12, alone and in different delivery vehicles, on the gut transit time, fecal short-chain fatty acids, and the composition of the gut microbiota of the study cohort.

## INTRODUCTION

The human gastrointestinal tract (GIT) harbors a diverse and dynamic community of microorganisms collectively termed the gut microbiota that contributes to the homeostasis of the gut and the biology of the host (1). The gut microbiota is estimated to contain approximately 40 trillion microbes, including hundreds of species of facultative and obligate anaerobes (2). The balance and composition of the gut microbiota can be altered by several factors such as medical interventions, age, genetics, environment, diet, and human health (3). Disturbed gut microbiota, also referred to as “dysbiosis” (4), has been linked to diseases such as obesity (5–8), inflammatory bowel disease (IBD) (9), and antibiotic-associated diarrhea (AAD) (10). Probiotics may help restore the microbiota of a disrupted GIT. Studies have shown that probiotic interventions significantly reduced the incidence of AAD in infants and children (11, 12).

Whole gut transit time (WGTT) refers to the time it takes for food to move from the mouth to the anus. Gut transit time (GTT) varies markedly among individuals, as well as within individuals, and maintaining a regular WGTT is essential for health and general well-being (13). Fecal short-chain fatty acid (SCFA) production has been associated with changes in gut microbiota and WGTT as a result of consuming probiotics, prebiotics, or symbiotics, but the findings are inconsistent (14). To better understand their relationship, it is important to study fecal SCFAs together with WGTT and the gut microbiota.

Probiotics, defined as “live microorganisms that, when administered in adequate amounts, confer a health benefit on the host” (15, 16), can be ingested as supplements in powder, capsule, or liquid form. Such supplements have shown potential benefits in the treatment and prevention of numerous diseases, including diarrhea, asthma, necrotizing enterocolitis, and allergies (17–21). As an alternative to delivery in supplements, probiotic organisms are often included as ingredients in fermented dairy products to produce functional foods, that is, foods providing health benefits beyond their nutritional value (22). Yogurt, for example, is a fermented milk product often considered a functional food. According to a survey conducted by Monroe Mendelsohn Research in 2001, two-thirds of primary care physicians who counsel patients about nutrition recommend consuming yogurt containing live and active cultures for health benefits (23). However, it is not clear whether probiotics delivered via dairy- and non-dairy-based matrices are equally effective, or whether one matrix is better in terms of benefiting the host’s gut microbiota, gut transit time, and fecal SCFA production. It is also uncertain how the yogurt fermentation process may affect the performance of probiotic organisms *in vivo*.

Probiotics marketed as nutritional supplements and found in functional foods are predominantly members of the genera *Bifidobacterium*, *Lactobacillus*, *Lactocaseibacillus*, *Ligilactobacillus*, *Lactiplantibacillus*, and *Limosilactobacillus*. *Bifidobacterium* species can be found in the gastrointestinal tract (GIT) as both autochthonous and allochthonous residents (24). Bifidobacterium animalis subsp. *lactis* (*BAL*), BB-12 (BB-12 is a trademark of Chr. Hansen A/S), the primary focus of this study, has been shown to improve bowel function, to support a healthy gut microbiota, and to improve immune function (25). However, the nature of the delivery vehicle (food matrix, tablets, or capsules)—including parameters such as water activity, pH, level and types of macronutrients (fat, protein, and carbohydrates), presence of organic acids, oxygen level, and the presence of other functional ingredients—may be important in determining how BB-12 will behave in a product and when ingested by the consumer. In a review paper, Sanders and Marco (26) pointed out that, “little is known about the food matrix and product formulation impacts on probiotic functionality even though such information is essential to scientific understanding and regulatory substantiation of health benefits.”

The objective of the present study was to evaluate the efficacy of delivering strain BB-12 via dairy (yogurt smoothie) or nondairy (capsule) vehicles in humans. Moreover, we determined the effects of these delivery vehicles in terms of modifying the gut microbiota, modulating gut transit time, and increasing SCFA production. The primary outcome of the project was WGTT, and the focus of the present study was to survey the gut microbiota of participants before and after different BB-12 interventions.

## RESULTS AND DISCUSSION

To test for the efficacy of delivery of *B. animalis* subsp. *lactis* BB-12, a randomized, four-period, crossover study performed in a partially blind manner was conducted. Thirty-six healthy adults were recruited and randomly assigned to four treatments: (i) control yogurt smoothie (YS), (ii) yogurt smoothie with strain BB-12 added prefermentation (PRE), (iii) yogurt smoothie with BB-12 added postfermentation (POST), and (iv) capsule containing BB-12 (CAP). Yogurt smoothies were manufactured using standard fermentation practices, and the concentration of BB-12 was measured through the shelf life of the products.

### Interventions.

Viable counts of strain BB-12 in all yogurt smoothies were evaluated weekly following production over the 30 days. Results from 27 batches revealed a significant difference in the population of BB-12 between the PRE and POST fermentation treatments immediately following manufacture (week 0) (initial counts of log_10_ 10.55 ± 0.12 CFU/serving and log_10_ 10.50 ± 0.14 CFU/serving, respectively). However, we believe this difference is not of significance in terms of product performance since the amount of BB-12 was above log 10 CFU/serving in both treatments. As expected, the population of BB-12 declined throughout the shelf life of the products with a faster decrease in the population for the POST treatment than in the PRE treatment. This trend continued, and by the end of the shelf life, the BB-12 concentration decreased significantly in both PRE (log_10_ 10.24 ± 0.13 CFU/serving) and POST (log_10_ 9.54 ± 0.25 CFU/serving) after 4 weeks’ storage (Table 1). BB-12 survived significantly better in PRE than in POST (*P < *0.001), indicating that BB-12 survives better when added before fermentation, possibly as a result of adaptation to the acidic environment (27). Overall, all BB-12 interventions remained at the targeted effective dose level (log_10_ 10 ± 0.5 CFU/serving) during shelf life.

**TABLE 1:** BB-12 concentration in yogurt smoothies during shelf life

Treatment^*a*^	BB-12 concentration (log_10_ CFU/serving)^*b*^ at the following time:				
	Week 0	Week 1	Week 2	Week 3	Week 4
PRE	10.55 ± 0.12 Aa	10.43 ± 0.13 Ba	10.42 ± 0.13 Ba	10.34 ± 0.13 Ca	10.24 ± 0.13 Da
POST	10.50 ± 0.14 Ab	10.16 ± 0.15 Bb	10.03 ± 0.20 Cb	9.77 ± 0.20 Db	9.54 ± 0.25 Eb

a PRE, yogurt smoothie with BB-12 added before fermentation; POST, yogurt smoothie with BB-12 added after fermentation.

b Data are presented as means ± standard deviations (SD) from 27 batches. Values in a column without a common lowercase letter are significantly different (*P* < 0.05). Values in a row without a common uppercase letter are significantly different (*P* < 0.05).

### Participant characteristics.

Baseline characteristics of the participants are shown in Table 2. Twenty-nine participants (18 females and 11 males) were included in the analyses, as they had completed at least one of the four intervention periods. Overall, participants were healthy young adults with a mean age of 28.1 ± 0.6 years. The average body mass index (BMI) was 24.1 ± 0.2 kg/m^2^: 17 (58.6%) participants were normal weight, 11 (37.9%) were overweight, and 1 (3.5%) was obese. Their blood pressure, waist circumference, fasting blood glucose, insulin, and C-reactive protein (CRP) levels were within the normal range (Table 2). Physical activity, as assessed from self-reported International Physical Activity Questionnaire (IPAQ) responses, indicated a median daily physical activity of 3.0 metabolic equivalents (METs) (range, 2.3 to 4.3 METs). The average daily total calorie intake of participants calculated from 3-day dietary recalls was estimated to be 2,241 ± 83 kcal. The daily intake of macronutrients, vitamins, minerals, and n-3 polyunsaturated fatty acids (PUFA), caffeine, and alcohol is also reported in Table 2.

**TABLE 2:** Demographic characteristics of participants before treatment (baseline)^*a*^

Characteristic	Value^*b*^ (*n* = 29)
Age (yr)	28.1 ± 0.6
Male, *n* (%)	11 (37.9%)
Body mass index (kg/m^2^)	24.1 ± 0.2
≤24.9	17 (58.6%)
25.0−29.9	11 (37.9%)
≥30	1 (3.5%)
Waist circumference (cm)	85.1 ± 0.6
Blood pressure (mm Hg)	
Systolic	107.6 ± 0.8
Diastolic	72.6 ± 0.6
Glucose (mg/dl)	86.6 ± 0.8
Insulin (mg/dl)	5.3 ± 0.4
hs-CRP (mg/liter)	2.0 ± 0.5
Physical activity (METs)^*c*^	3.0 (2.3–4.3)
Dietary intake^*c*^	
Total calories (kcal/day)	2,241 ± 83
Carbohydrate (g/day)	284.6 ± 10.9
Protein (g/day)	90.2 ± 3.7
Fat (g/day)	83.8 ± 3.4
Vitamin C (mg/day)	67.8 ± 4.8
Vitamin D (IU/day)	98.5 ± 11.2
Vitamin E (mg/day)	3.4 ± 0.3
Iron (mg/day)	14.4 ± 0.8
Selenium (μg/day)	46.0 ± 4.5
Zinc (mg/day)	5.7 ± 0.4
n-3 PUFA (g/day)	0.6 ± 0.1
Caffeine (mg/day)	75.3 ± 10.2
Alcohol consumption (g/day)	2.6 ± 0.9

a Shared results with collaborators in the project.

b Values are presented as means ± standard errors of the means (SEM) or *n* (%) or median (range).

c Physical activity and dietary intake were assessed from self-reported responses to IPAQ and 3-day dietary recall records, respectively.

### Compliance.

All stool DNA samples were tested for compliance using 16S rDNA-based subspecies-specific PCR. A total of 73 out of 78 samples were *B. animalis* subsp. *lactis* (*BAL*) positive after BB-12-containing interventions, while all the fecal samples were *BAL* negative after receiving the control intervention when the corresponding baselines were negative. Four participants were *BAL* positive before treatment (baseline) and throughout the study regardless of treatments, suggesting that *BAL* was autochthonous to these individuals.

### Primary outcome.

Only 27 participants were included in primary outcome analysis because a few data points could not be retrieved from the data receiver due to technical difficulties. Results of the analysis indicated no treatment effect on gut transit times (data not shown). In the present study, participants had a wide range of WGTT (7.13 h to 128.58 h), colonic transit time (CTT) (0.5 h to 122.25 h), small bowel transit time (SBTT) (1 h to 19.02 h), and gastric emptying time (GET) (0.98 h to 18.83 h). Subjects with extremely short CTT (0.5 h) might have had diarrhea. The variability of responses among individuals made it difficult to detect a treatment effect, if there was any. In agreement with a previous study (28), males in the present cohort had shorter CTT (*P* = 0.0098), WGTT (*P = *0.0036), and GET (*P* < 0.0001) than females but exhibited no difference in SBTT (*P = *0.3). There was a significant correlation between the blue dye and SmartPill measurements (Spearman rho = 0.67, *P* < 0.0001), which suggests that the blue dye method remains a useful screening tool for GTT in healthy individuals. However, because the SmartPill is a more objective measure of GTT, the results for WGTT are likely better estimates of actual transit time.

In previous work, the effect of a BB-12 intervention (delivered in fermented milk, capsule, or fermented cereal) on host bowel movement was studied in both healthy subjects and subjects with functional bowel symptoms, and promising improvements were observed (29–31). A large clinical trial with 1,248 subjects performed in eight centers in Europe reported a treatment effect of strain BB-12 (delivered in capsule) on average defecation frequency (*P* = 0.0065) (32), despite the fact that the placebo group also had increased average defecation frequency compared to baseline. Several factors may explain the lack of treatment effect on GTT, as well as the discrepancy between our results and previous studies. First, the present study had a relatively small sample size, which makes it difficult to detect a small treatment effect within a highly variable data set. Second, the cohort studied is a generally healthy group of individuals, with whom there is only limited room for improvement in terms of bowel transit time. Finally, this study took direct measurements of the GTT using a wireless motility capsule in contrast to other studies that employed more subjective defecation frequency questionnaires.

### Secondary outcomes. (i) Characteristics of the fecal microbiota of the participants.

Illumina sequencing of the 161 fecal samples generated over 2.6 million total reads. After removing samples with low quality and poor compliance, about 2.4 million sequences from 147 samples were used for data analyses. Overall, 10 phyla and 109 genera were identified in the participants. *Firmicutes*, *Bacteroidetes*, *Actinobacteria*, and *Proteobacteria* accounted for >98% of the sequences at the phylum level. The predominant phyla and genera identified (represents > 0.1% of the sequences) among treatment groups are shown in Table 3. No difference at the phylum or genus level was detected among treatment groups, with the exception of a significantly higher percentage of Streptococcus in their fecal microbiota after the participants consumed control yogurt smoothie compared to consuming capsule (*P* = 0.01). All yogurt smoothies tended to have a higher percentage of Streptococcus compared to baseline, capsule, and final washout. This is likely due to the presence of the high level (log_10_ 11.4 CFU/day) of S. thermophilus in the yogurt interventions.

**TABLE 3:** Predominant fecal bacterial phyla and genera present in healthy adults before and after consuming BB-12-containing interventions in a crossover study

Phylum and genus	% of sequences^*a*^							
	Baseline	YS	POST	PRE	CAP	Final washout	SEM	*P* value
*Firmicutes*	82	82	80	79	82	79	0.76	0.58
* Blautia*	8.4	8.3	7.9	6.8	8.2	7.3	0.35	0.77
* Faecalibacterium*	5.2	6.3	5.4	6.4	6.2	6.9	0.34	0.69
* Ruminococcus*	4.9	4.1	5.4	5.4	5.7	5.3	0.33	0.78
* Coprococcus*	2.7	3.3	3.3	2.8	3.1	3.1	0.13	0.74
* Roseburia*	1.7	1.8	1.8	1.4	2.0	2.6	0.19	0.62
* Lachnospira*	1.2	1.8	2.1	1.5	1.6	1.7	0.13	0.21
* Dialister*	1.0	1.1	1.1	1.3	1.4	0.8	0.12	0.77
* Dorea*	1.0	1.1	0.89	0.67	1.0	0.9	0.05	0.20
**Streptococcus**	**0.59 AB**	**1.04 A**	**0.89 AB**	**0.73 AB**	**0.45 B**	**0.38 B**	**0.06**	**0.01**
* Clostridium*	0.56	0.77	0.55	0.57	0.70	0.81	0.06	0.76
* Oscillospira*	0.83	0.51	0.61	0.69	0.57	0.56	0.05	0.46
* Enterococcus*	0.40	0.40	0.41	0.42	0.35	0.37	0.02	0.81
* Lactobacillus*	0.22	0.29	0.30	0.32	0.48	0.64	0.07	0.54
* Lachnobacterium*	0.29	0.28	0.08	0.47	0.19	0.33	0.07	0.72
* Anaerostipes*	0.23	0.18	0.22	0.23	0.31	0.20	0.02	0.65
* Turicibacter*	0.21	0.17	0.13	0.08	0.12	0.24	0.02	0.45
* Megasphaera*	0.08	0.07	0.10	0.21	0.10	0.13	0.03	0.79
*Bacteroidetes*	12	13	14	14	12	14	0.64	0.81
* Bacteroides*	8.3	8.9	9.9	9.2	7.9	10.3	0.53	0.81
* Parabacteroides*	0.92	1.0	1.1	1.0	1.0	1.1	0.08	0.96
* Prevotella*	0.44	0.46	0.38	0.81	0.28	0.28	0.14	0.91
*Actinobacteria*	3.7	2.8	3.6	4.4	3.7	3.7	0.36	0.72
* Bifidobacterium*	3.5	2.6	3.3	4.2	3.4	3.5	0.36	0.71
* Collinsella*	0.11	0.12	0.11	0.12	0.13	0.14	0.01	0.96
*Proteobacteria*	1.5	1.7	1.5	1.8	1.3	1.5	0.08	0.49
* Sutterella*	0.22	0.30	0.27	0.42	0.30	0.40	0.03	0.34
* Citrobacter*	0.22	0.20	0.21	0.37	0.22	0.24	0.03	0.32
* * Haemophilus	0.13	0.23	0.10	0.12	0.05	0.14	0.03	0.64
*Verrucomicrobia*	0.19	0.11	0.24	0.18	0.15	0.36	0.03	0.94
* Akkermansia*	0.19	0.11	0.24	0.18	0.15	0.36	0.00	0.41

a Values are presented as means with pooled SEMs (*n* = 147). Values in a row without a common letter are significantly different (*P* < 0.05).

The *Firmicutes*/*Bacteroidetes* (F/B) ratio (median, 6.79) of the present study cohort is at the high end compared with the results of the Human Microbiome Project (HMP) (33). However, the results are comparable to other studies (6, 34, 35). The F/B ratio is known to vary dramatically among populations, as well as between age groups (36).

**FIG 1:**
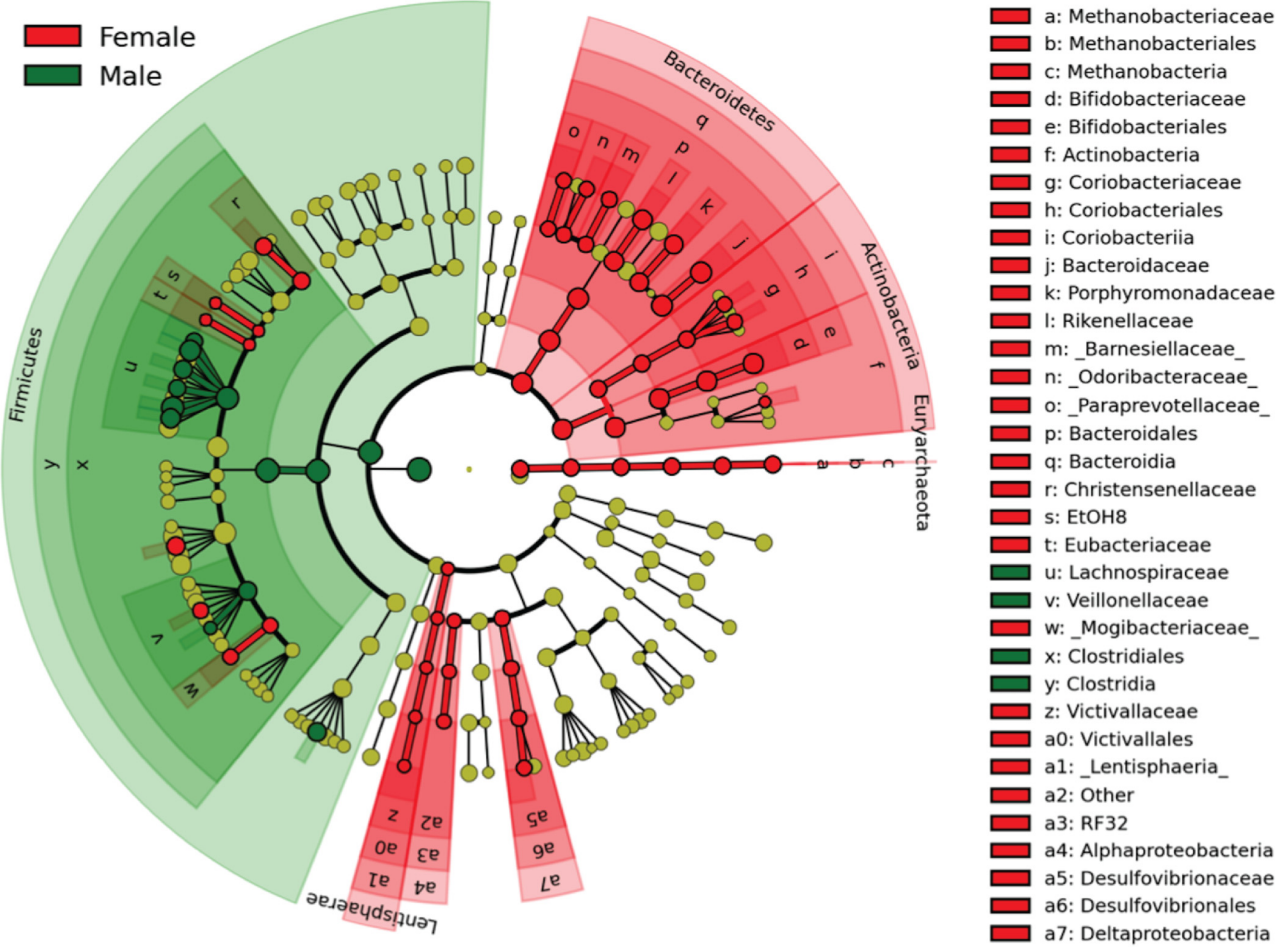
Taxonomic cladogram of LDA effect size comparing the relative abundance of taxa in males and females. Significantly discriminant taxon nodes are colored and branch areas are shaded according to the highest-ranked variety for that taxon. For each taxon detected, the corresponding node in the taxonomic cladogram is colored according to the highest-ranked group for that taxon. If the taxon is not significantly different between groups, the corresponding node is colored yellow (66).

Comparisons at various taxonomic levels between each treatment period and baseline were performed using linear discriminant analysis effect size (LEfSe). Overall, there was little difference between any treatment and baseline, other than overrepresented Streptococcus in yogurt groups (data not shown). Interestingly, a gender difference was observed in the same data set (Fig. 1). Females had significantly greater abundance of *Paraprevotella*, *Butyricimonas*, *Parabacteroides*, *Bacteroides*, *Collinsella*, *Bifidobacterium*, *Varibaculum*, *Methanobrevibacter*, and *Oscillospira*, while males had significantly higher percentages of *Anaerostipes*, *Blautia*, *Dorea*, *Lachnobacterium*, and *Roseburia*. Gender differences in gut microbiota have also been observed in an animal study (37) and in other human studies (38–41), although some studies have reported no difference or a modest association (33). Gender differences in gut microbiota have been proposed to be influenced by sex hormone levels (40), gender-based differences in the immune system (41), and differences in dietary patterns (42). Coincidently, a gender difference in responsiveness of CD14^+^ HLA-DR^+^ cells to the interventions has been previously observed (43), where the percentage of CD14^+^ HLA-DR^+^ cells was increased only in the male participants following consumption of all yogurt-containing treatments compared with baseline.

In summary, only limited compositional changes were detected in participants’ fecal microbiota after consuming BB-12-containing capsules or yogurt smoothies compared to baseline. Yogurt interventions appeared to result in elevated relative abundance of Streptococcus in host fecal microbiota, especially in female participants, but gender appeared to be a more significant factor in shaping the gut microbiota than the treatments, at least in this study cohort.

### (ii) Diversity of the participants’ fecal microbiota.

**FIG 2:**
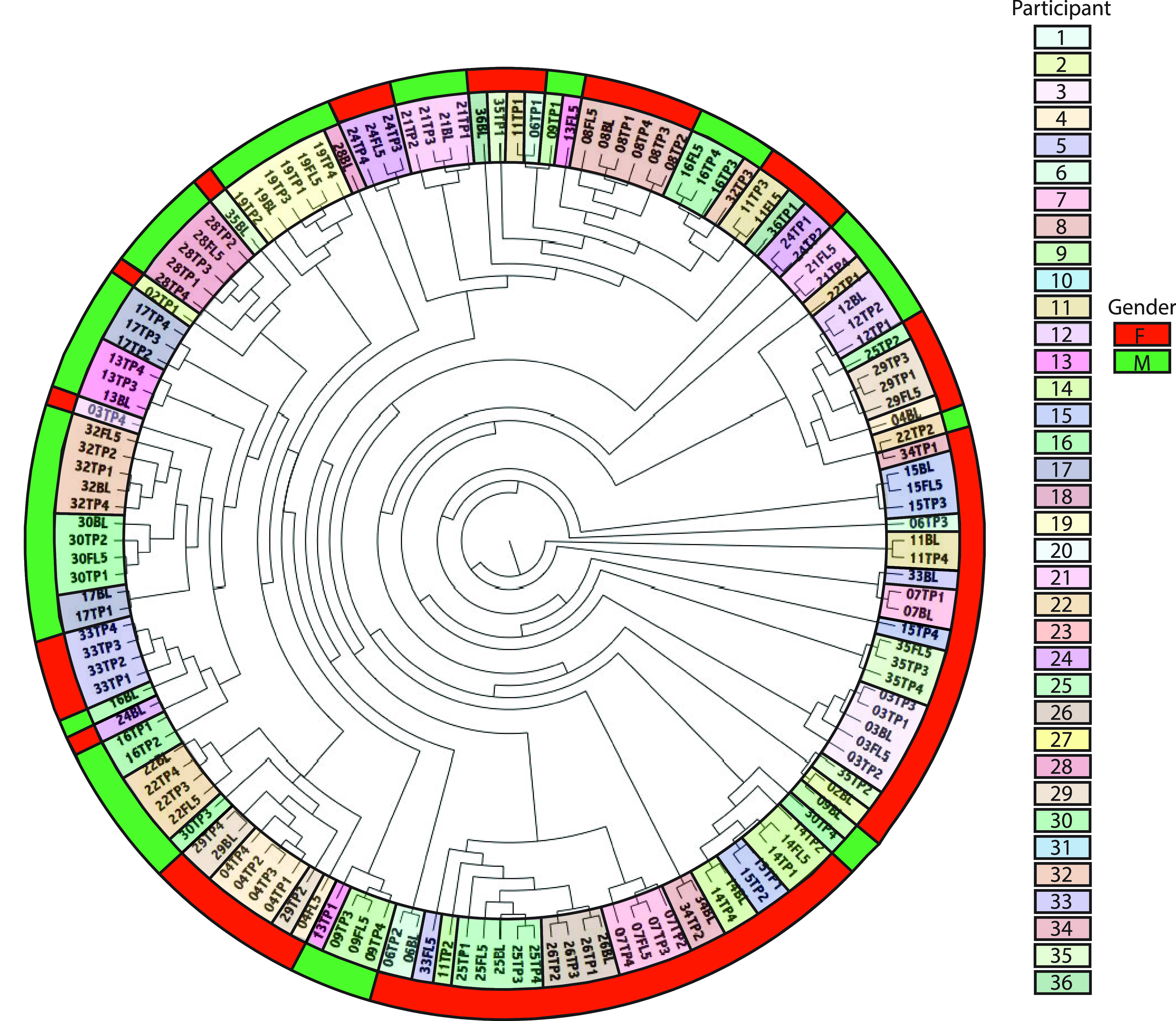
UPGMA tree based on weighted UniFrac distance (beta diversity) demonstrating the hierarchical relationships between the fecal samples. The code is participant’s study identifier (ID) followed by treatment period (i.e., TP1 is the first treatment period, BL is baseline, FL5 is final washout). The inside color bar indicates participant ID, and the outside color bar indicates the gender (female [F] or male [M]) of each participant. The data indicate that samples from each individual tend to cluster together regardless of treatment.

There were no significant differences in alpha diversity indices (Chao1 richness, Simpsons diversity, and Shannon evenness) between treatment groups or between genders (data not shown). A definitive stratification according to treatment group was not evident on UPGMA (unweighted pair group method with arithmetic mean) tree, but samples from each individual tended to cluster together (Fig. 2). Permutational multivariate analysis of variance (PERMANOVA) also confirmed the similarity between treatments (*R*_ANOSIM_ = −0.041, *P = *1.0; *R*^2^_ADONIS_ = 0.024, *P = *1.0) and dissimilarity between individuals (*R*_ANOSIM_ = 0.925, *P = *0.001; *R*^2^_ADONIS_ = 0.433, *P = *0.001). In this case, the results indicated that individual characteristics played a bigger role than treatments in shaping the host gut microbiota. This is supported by previous research on both animals (44) and humans (45). Specifically, Goodrich et al. studied over 1,000 stool samples from 416 pairs of twins (45). They found that fecal microbiota were more similar overall within individuals (resampled) than between unrelated individuals (*P* < 0.001) and were also more similar within twin pairs than unrelated individuals (*P < *0.009). Moreover, monozygotic twin pairs had a more similar gut microbiota than dizygotic twin pairs (*P* = 0.032) (45).

**FIG 3:**
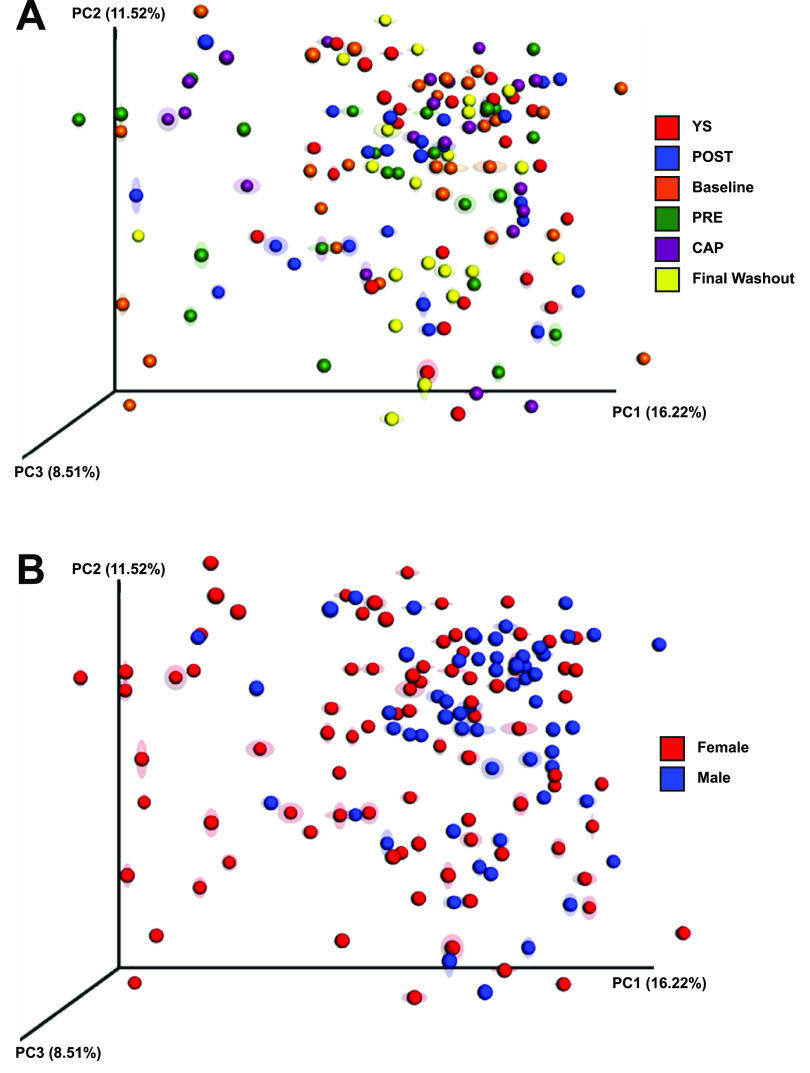
Weighted UniFrac distance PCoA of bacterial communities with jackknife support grouped by treatment and gender. There were no patterns of clustering when samples were colored by treatments, while samples tend to cluster based on gender, suggesting a gender difference.

A visible difference in community structure between treatments was not noted on the principal coordinate analysis (PCoA) plot; however, samples tended to cluster when grouped by gender (Fig. 3). Statistical analyses revealed that community membership was different between males and females (*R*_ANOSIM_ = 0.096, *P = *0.002; *R*^2^_ADONIS_ = 0.021, *P = *0.001), although only a small percentage of differences can be explained by the data set. Other metadata (i.e., glucose, high/low-density lipoprotein, triglycerides, C-reactive protein, tumor necrosis factor alpha, and interferon gamma) reported elsewhere (46, 47) were screened for possible associations with the host gut microbiota data. A number of statistically significant differences were found (see Table S1 in the supplemental material). However, further studies are needed to validate these relationships.

10.1128/mSphere.00084-21.1 TABLE S1 Statistical analyses of the weighted UniFrac distance (beta diversity) when grouped by the metabolic and immune parameters measured as described in references 46 and 47. Abbreviations: CRP, C-reactive protein; TC:HDL-C, total cholesterol (TC) to high-density lipoprotein cholesterol (HDL-C) ratio; WC, waist circumference; TG, triglycerides; LDL-C, low-density lipoprotein cholesterol; DCs, dendritic cells; IFN-γ, interferon gamma; TNF-α, tumor necrosis factor alpha; IL-2, interleukin-2. Download Table S1, DOCX file, 0.01 MB. Copyright © 2021 Ba et al. 2021 Ba et al. https://creativecommons.org/licenses/by/4.0/ This content is distributed under the terms of the Creative Commons Attribution 4.0 International license.

Taken together, the diversity results are not completely surprising. Participants in the present study cohort were healthy individuals, and it is well understood that a healthy gut microbiota is stable and resilient (33, 48, 49). Although consuming a high-fat and low-fiber or low-fat and high-fiber diet for 10 days can induce statistically significant changes in the gut microbiota, these changes in species and gene content are small compared with baseline variations that occur between individuals (50).

In previous work, McNulty et al. (51) repeatedly sampled seven healthy adult female monozygotic twin pairs (aged 21 to 32 years, BMI of 20 to 25 kg/m^3^) 4 weeks before, 7 weeks during, and 4 weeks after consumption of a commercially available fermented milk product (8 oz/day) containing a consortium of *BAL* strain CNCM I-2494 (3.2 × 10^7^ CFU/g), Lactobacillus delbrueckii subsp. *bulgaricus* strains CNCM I-1632 and I-1519 (6.3 × 10^7^ CFU/g), Lactococcus lactis subsp. *cremoris* strain CNCM I-1631, and Streptococcus thermophilus strain CNCM I-1630. They found the species and gene content of the twins’ gut microbial communities remained stable and were not appreciably perturbed by consuming the intervention. However, another study of mice reported that introducing the fermented milk product strains resulted in marked changes in metabolic pathways related to carbohydrate processing, although the proportional representation of their gut microbiota acquired from their human donors remained the same (51). The authors suggested that analyses of the bacterial species and gene content of the gut microbiota/microbiome may not be informative biomarkers for understanding whether or how the interventions may have affected microbial community properties.

### (iii) Fecal bifidobacterial distribution.

**FIG 4:**
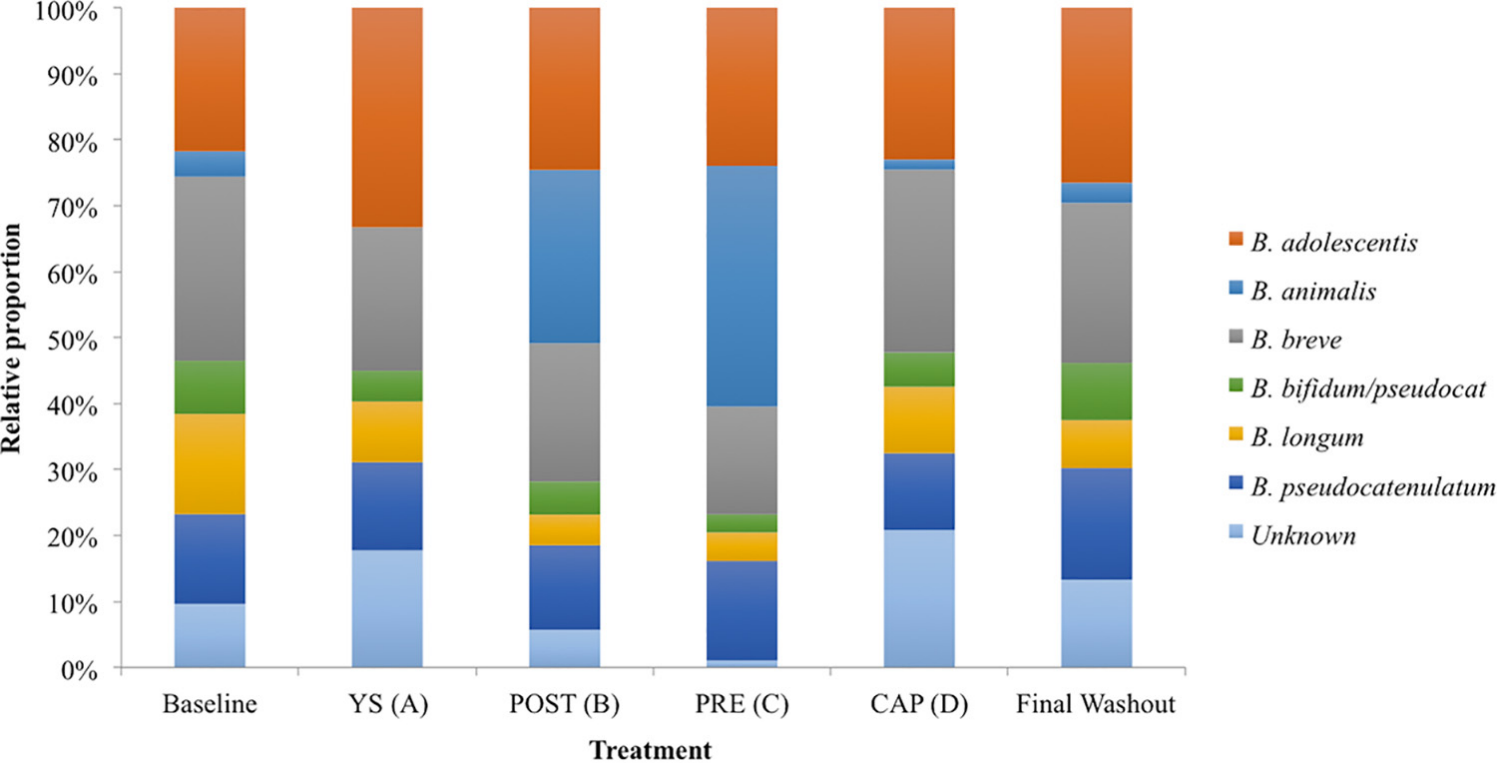
Relative proportion of *Bifidobacterium* species in the stool DNA samples before treatment (baseline), after each treatment, and after final washout as determined by *Bif*-TRFLP (AluI).

To further explore the effect of the study interventions on the host gut bifidobacteria at the species level, stool DNA samples were subjected to *Bifidobacteria* terminal restriction fragment length polymorphism (*Bif*-TRFLP) (52). Participants had a significantly higher percentage of B. animalis as a portion of the total bifidobacteria in their feces after consuming the two yogurt smoothies containing BB-12 (PRE and POST) compared to baseline, other interventions (YS or CAP), and final washout (*P* < 0.0001) (Fig. 4). There was no significant difference between PRE and POST yogurt smoothie treatments. It appears that the BB-12-containing yogurt smoothie resulted in higher relative abundance of *B. animalis* in the stool samples than capsules. This may be due to the buffering capacity of milk proteins that could protect strain BB-12 when passing through the acidic conditions of the stomach (53). Care should be taken when extrapolating this finding, because the results presented here are not absolute concentrations. Moreover, it is unclear what the physiological consequences are when the host has a higher level of *B. animalis* in their feces.

The physiological effect of the delivery matrix on the performance of probiotics has been previously observed in an animal study. Lee et al. (54) fed dextran sulfate sodium (DSS)-induced ulcerative colitis mice, with a wild-type strain and two mutant (DltD^−^ and RecA^−^) probiotic strains of Lacticaseibacillus casei (2 × 10^7^ CFU/feeding) in milk or a nutrient-free buffer prior to and during administration of DSS for 15 consecutive days. Live *Lb. casei* cells in stool samples were recovered using selective De Man, Rogosa, and Sharpe (MRS) medium. A disease activity index (DAI) was calculated by percent total weight loss (before/after DSS treatment), histology score, the presence of blood in stools, and stool consistency. This study showed that mice fed with *Lb. casei* in milk had lower DAI than those fed with *Lb. casei* in nutrient-free buffer, milk only, and mutant in either milk or buffer, suggesting that milk might be the preferred delivery matrix for certain probiotic strains. Additional studies are needed to evaluate other strains, other delivery matrices, and different disease conditions. Moreover, the underlying mechanisms need to be further investigated.

### (iv) Predominant fecal SCFAs correlate with GTTs.

Short-chain fatty acids are important for maintaining a healthy gastrointestinal (GI) environment as they promote the growth and differentiation of epithelial cells and provide energy to colonocytes (55). Fecal SCFA concentrations were measured before treatment (baseline), after each treatment, and after final washout. The most abundant SCFAs detected in human fecal samples were acetic acid (30.7%), propionic acid (21.3%), and butyric acid (31.8%), and there was no difference in SCFA levels among treatments. Previous studies have reported that the ratio of SCFAs in human fecal samples is 60:20:20 for acetate-butyrate-propionate (56, 57). The discrepancy between our study could be due to differences in analytical techniques (such as extraction or quantification), as the ratio was consistent throughout treatments and participants. In this study, the concentrations of SCFAs were significantly correlated (Table 4). Moreover, males had a higher butyric acid concentration than females in this study cohort (median of 908 μg/g versus 687 μg/g; *P* = 0.0233) in agreement with a previous study (58). High interindividual variations were observed in the content of fecal SCFAs (relative standard deviation, 52% to 118%).

**TABLE 4:** Spearman correlations of GTT and fecal SCFAs^*a*^

Variable or SCFA	Statistical parameter^*b*^	Spearman correlation or *P* value of variable and SCFA									
		WGTT	CTT	SBTT	GET	Acetic acid	Propionic acid	Isobutyric acid	Butyric acid	Isovaleric acid	Valeric acid
CTT	Sr	0.97									
	P	<0.001									
SBTT	Sr	0.26	0.12								
	P	0.003	0.163								
GET	Sr	0.21	0.12	−0.06							
	P	0.018	0.175	0.479							
Acetic acid	Sr	−0.19	−0.19	0.05	−0.29						
	P	0.036	0.033	0.566	0.001						
Propionic acid	Sr	−0.25	−0.25	0.03	−0.27	0.68					
	P	0.005	0.004	0.763	0.003	<0.001					
Isobutyric acid	Sr	0.04	0.06	0.03	−0.16	0.11	0.29				
	P	0.697	0.492	0.714	0.072	0.224	0.001				
Butyric acid	Sr	−0.21	−0.21	0.06	−0.36	0.86	0.73	0.23			
	P	0.021	0.016	0.488	<0.001	<0.001	<0.001	0.008			
Isovaleric acid	Sr	0.07	0.10	0.01	−0.1	−0.06	0.18	0.971	0.09		
	P	0.407	0.285	0.925	0.250	0.533	0.038	<0.001	0.311		
Valeric acid	Sr	−0.1	−0.12	0.20	−0.12	0.49	0.54	0.64	0.51	0.55	
	P	0.261	0.183	0.026	0.087	<0.001	<0.001	<0.001	<0.001	<0.001	
Caproic acid	Sr	0.07	0.08	0.07	−0.01	0.24	−0.03	0.21	0.16	0.14	0.39
	P	0.417	0.350	0.407	0.897	0.006	0.759	0.018	0.076	0.107	<0.001

a Abbreviations: GTT, gut transit time; SCFAs, short-chain fatty acids.

b Sr, Spearman rho value; P, *P* value.

Since fecal SCFAs only account for less than 5% of the total SCFAs produced in the colon (54, 57), the effectiveness of strain BB-12 in promoting SCFA production in the large intestine remains unclear. Previous studies reported inconsistent results. In an early study of 16 patients with ileal pouch-anal anastomosis for ulcerative colitis, no difference in SCFA content was observed after consumption of 500 ml of fermented milk (Cultura) containing >10^8^ CFU/ml of both Lactobacillus acidophilus (LA-5) and *BAL* (BB-12) or heat-treated Cultura for 1 week compared to baseline or between groups (59). On the other hand, a more recent study demonstrated that consumption of yogurt containing log_10_ 9.72 CFU of *BAL* LKM512 per day for 4 weeks tended to increase fecal butyrate concentration in patients with atopic dermatitis compared to baseline (*P* = 0.08) (60).

To further explore possible relationships between GTTs and fecal SCFA concentrations, correlation analyses were conducted. Gut transit times were negatively correlated with predominant SCFAs, but valeric acid was positively correlated with SBTT (Table 4). A previous study on the effect of GTT rate on fecal SCFA concentration showed negative correlations between GTT and fecal SCFAs, which was attributed to physiological factors of the participants (61).

### Limitations.

One of the major limitations of the present study was the inability to recruit enough participants with significantly delayed WGTT (>60 h). As a result, volunteers with slightly delayed WGTT (>24 h) were recruited, leaving little room for the intervention to have a significant effect. Moreover, performing only one measurement before treatment (baseline) did not seem to reflect the true gut transit status, as each subject’s WGTT tended to vary significantly from one day to another. Finally, the sequencing technique employed had a low resolution that can only detect down to the genus level. This made it impossible to explore treatment effect at the species level, although supplemental methods were used to measure species of interest.

### Conclusions.

The present study evaluated the effect of the probiotic *BAL* BB-12 alone (capsule) or when incorporated into yogurt smoothies before or after yogurt fermentation on the GTTs, fecal SCFA concentrations, the composition of the gut microbiota, and the bifidobacterial profile of young healthy adults. No significant treatment effects on the GTTs, fecal SCFAs, or the gut microbiota were detected due to the large interindividual and intraindividual variations observed. A significant gender effect was observed when comparing the gut microbiota of the cohort of the present study. Interestingly, the two BB-12-containing yogurt smoothies (PRE and POST) resulted in significantly higher percentage of *B. animalis* as a fraction of total bifidobacteria compared to baseline, to the BB-12-free yogurt smoothie (YS), the capsule (CAP), and final washout when analyzed by *Bif*-TRFLP. No difference was detected between PRE and POST addition of BB-12. Although *Bif*-TRFLP could not measure viability, the finding of the present study may shed light on the subtle effect of probiotic interventions on the gut microbiota at the species level. Further studies are warranted to study other bacterial groups at the species level, more importantly, to clarify the impact of these differences on human health.

## MATERIALS AND METHODS

### Study design.

**FIG 5:**
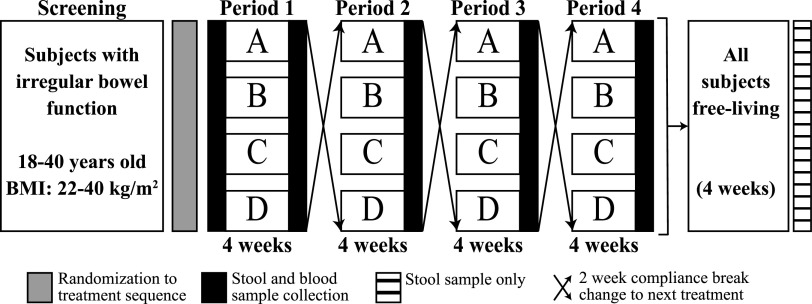
Schematic diagram for randomization design. The treatments are shown in boxes as follows: A, yogurt smoothie without BB-12 (YS); B, yogurt smoothie with BB-12 added postfermentation (POST); C, yogurt smoothie with BB-12 added prefermentation (PRE); D, BB-12-containing capsule (CAP).

This clinical trial was a randomized, four-period, crossover study of free-living subjects performed in a partially blind manner. The study design scheme is shown in Fig. 5. The detailed clinical aspects of this study have been reported elsewhere (46, 47). Briefly, measurements taken during the baseline visit and after each treatment were anthropometric assessment (age and gender at the baseline visit only, BMI, waist circumference), biochemical parameters (fasting serum glucose, insulin, and high-sensitivity CRP [hs-CRP]), a physical activity questionnaire (self-reported IPAQ), and an immune endpoint assessment. Then each participant began the intervention phase as specified by the randomization order. The four treatments were YS (yogurt smoothie without BB-12) (treatment A), POST (yogurt smoothie with BB-12 added after fermentation) (treatment B), PRE (yogurt smoothie with BB-12 added before fermentation) (treatment C), and CAP (BB-12-containing capsule (treatment D). Each treatment period lasted 4 weeks, and a 2-week wash-out compliance break was scheduled between treatment periods.

### Interventions.

The control and BB-12 (Chr. Hansen A/S, Hørsholm, Denmark) interventions were strawberry-flavored yogurt smoothies developed and manufactured at The Pennsylvania State University. The starter culture used was YF-L702 (Chr. Hansen A/S, Hørsholm, Denmark), a commercial blend of Streptococcus thermophilus and Lactobacillus delbrueckii subsp. *bulgaricus* (59). Dry ingredients were mixed into the milk, pasteurized at 84.4°C for 41 s, and homogenized at 2,000 lb/in^2^ in the first stage and at 500 lb/in^2^ in the second stage. Yogurt mix was then heat treated at 85°C for 30 min, cooled to 43.3°C, inoculated with YF-L702, and split in two portions. One portion remained BB-12 free, while another portion was inoculated with BB-12 (PRE). Both yogurt mixes were fermented until a pH of 4.6 was reached. At this time, a prepared mixture containing strawberry, pectin, corn syrup solids, sugar, and water was added and blended into the yogurt until uniform. After addition of the slurry, the BB-12-free yogurt smoothie was further split into two parts; one part was inoculated with BB-12 (POST), while the other part remained BB-12-free (YS). Finally, each of the products was rehomogenized to produce a drinkable yogurt. The three products were identical in chemical and textural characteristics except for the addition of BB-12 and the timing of BB-12 addition.

To verify the viable count of Bifidobacterium animalis subsp. *lactis* (*BAL*) in the yogurt smoothies, both control and BB-12 products were analyzed immediately after manufacture and weekly during shelf life. To determine cell counts, suitable dilutions were pour plated on MRS-NNLP agar (62) followed by anaerobic incubation at 37°C for 72 h. Colonies counted as *BAL* were randomly picked and confirmed to be *BAL* by PCR using subspecies-specific primers (63).

During the yogurt smoothie treatment phases, participants consumed one 240-g serving of yogurt per day. Study interventions contained between log_10_ 10 ± 0.5 × 10^9^ or 3.16 × 10^9^ and 3.16 × 10^10^ CFU of strain BB-12 per serving. During the CAP treatment phase, participants ingested one capsule per day. This CAP was specifically designed by Chr. Hansen A/S to deliver log_10_ 10 ± 0.5 CFU of BB-12/capsule, which was confirmed by viable counting as described for the yogurt smoothies throughout the study. Participants were instructed to avoid consuming any other food or supplements containing probiotic bacteria, such as commercial yogurt, smoothies, and probiotic capsules or tablets, during each 4-week intervention phase. They were also asked not to change their habitual diets for the course of the study and to maintain their body weight.

### Participants.

The primary outcome of the study, whole gut transit time (WGTT), was used for the sample size calculation. Specifically, the calculation was based on the data from a previous study (64) and indicated that 28 subjects were required to identify a mean difference of 6 h in WGTT. Considering the possibility of withdrawal from the study and potential lack of compliance, 36 healthy volunteers (18 to 40 years of age) with delayed WGTT (≥24 h) were recruited. Of these volunteers, 29 finished at least one treatment period. Detailed exclusion criteria are described elsewhere (46). All subjects were nonsmoking, normotensive, and not diagnosed with any chronic medical conditions. Prior to the start of the trial, all participants signed an informed consent form. The study was approved by the Institutional Review Board of The Pennsylvania State University (University Park, PA) under IRB 35111. This trial was registered at ClinicalTrials.gov as NCT01399996.

### Primary outcome measures.

The primary outcome WGTT was measured using a modified blue food dye method (65) and using a wireless mobility capsule, SmartPill (Given Imaging, Duluth, GA) at baseline, and only SmartPill after each treatment. Unlike the blue dye method which only measures WGTT, the SmartPill measured regional gut transit times (GTTs) including gastric emptying time (GET), small bowel transit time (SBTT) and colonic transit time (CTT) along with pH, temperature, and pressure throughout the gastrointestinal tract (GIT).

### Secondary outcome measures.

The secondary outcomes were to evaluate the effect of BB-12 interventions on the participants’ fecal microbiota (both composition and diversity) and to detect any shift in fecal SCFA concentrations.

### (i) Stool DNA extraction.

Fecal samples were collected by participants and stored at home freezers until transported to the Penn State Clinical Research Center on ice before treatment (baseline), after each treatment, and after final washout. Stool DNA was extracted using a MOBIO PowerSoil DNA isolation kit (catalog no. 12888; Qiagen, Carlsbad, CA) according to the manufacturer’s protocol. Isolated genomic DNA was stored at −80°C prior to analysis.

### (ii) Compliance.

Compliance was assessed by a *BAL* subspecies-specific PCR method (63). Detection of *BAL* in the feces of the BB-12 groups and the absence of *BAL* in the feces of the baseline group and the control smoothie group were taken as evidence of compliance. Results showing the presence of *BAL* in the control or baseline, or absence of *BAL* in the BB-12 groups, or missing fecal samples, were considered to indicate noncompliance.

### (iii) 16S rDNA amplicon Illumina sequencing.

DNA samples were prepared as previously described (66) with the following modifications. Universal primers F515 (5′-*NNNNNNNN***GT**GTGCCAGCMGCCGCGGTAA-3′) and R806 (5′-GGACTACHVGGGTWTCTAAT-3′), with the forward primer modified to contain an 8-nucleotide (nt) barcode [italicized poly(N) section of the primer above] and 2-nt linker sequence (boldface portion) at the 5′ end, were used to amplify the V4 region of the 16S rRNA gene. PCR mixtures contained 5.0 μl of 2 × GoTaq Green Master Mix (Promega, Madison, WI), 0.4 μl of 25 mM MgCl_2_, 2.4 μl of water, 0.2 μl of reverse primer (10 mM final concentration), 1.0 μl of forward primer (2 mM final concentration), and 1.0 μl of genomic DNA. Reactions were held at 94°C for 3 min to denature the DNA, with amplification proceeding for 25 cycles, with 1 cycle consisting of 94°C for 45 s, 50°C for 60 s, and 72°C for 90 s; a final extension of 10 min at 72°C was included to ensure complete amplification. The PCR products were purified using QIAquick PCR purification kit (catalog no. 28106; Qiagen, Valencia, CA). A sequencing library was created by combining equimolar ratios of amplicons from individual samples. The composite sample was sequenced at the DNA Technologies Core Facility of the University of California, Davis, on an Illumina Genome Analyzer II sequencing platform.

### (iv) *Bifidobacteria* terminal restriction fragment length polymorphism assay.

The *Bifidobacteria* terminal restriction fragment length polymorphism (*Bif*-TRFLP) assay was performed based on the method described by Lewis et al. (52). Briefly, stool DNA samples were amplified for bifidobacterial 16S rRNA gene. The PCRs were carried out in a mixture (50 μl) that contained 1 μl of genomic DNA, 25 μl of 2× Promega GoTaq Green Master Mix (Promega, Madison, WI), 20 μl of nuclease-free water, 1 μl of each primer (NBIF389, 5′-[HEX]-GCCTTCGGGTTGTAAAC-3′, 10 μM; NBIF1018REV, 5′-GACCATGCACCACCTGTG-3′, 10 μM), and 2 μl of MgCl_2_ (25 mM). The reaction conditions were 95°C for 5 min, followed by 30 cycles, with 1 cycle consisting of 95°C for 1 min, 52°C for 1 min, and 72°C for 1 min. A final extension at 72°C for 5 min was allowed following the cycles, and then the samples were stored at 4°C prior to analysis. PCR products were purified using the QIAquick PCR purification kit (catalog no. 28106; Qiagen, Valencia, CA).

A portion (8 μl) of the purified DNA was digested with two restriction enzymes (AluI [Thermo Fisher Scientific, Waltham, MA] and HaeIII [New England BioLabs Inc., Ipswich, MA]) in separate reactions, both of which involved 1 μl of enzyme (10 U/μl) in a 10-μl reaction mixture for 3 h at 37°C. Then, the enzymes were heat inactivated at 80°C for 20 min, and samples were stored at 4°C. Next, 1 μl of the digested mixture (diluted 1:20 in elution buffer) was submitted for fragment analysis on an ABI 3730 Capillary Electrophoresis Genetic Analyzer (Applied Biosystems, Grand Island, NY). The molecular size markers used were the ROX 50-500 size standards (Gel Company Inc., San Francisco, CA). The results were read using Peak Scanner software v1.0 (Applied Biosystems, Grand Island, NY). Detailed data processing is described in the original article (52).

### (v) Microbiome sequence data analysis.

The data analysis pipeline used was modified from a previous study (67). Briefly, QIIME software package (68) was used to analyze the results of the Illumina sequencing run. Raw Illumina fastq files were first demultiplexed and quality filtered. Reads were truncated after a maximum number of three consecutive low-quality scores (<1e^−5^), and any read containing one or more ambiguous base calls was discarded. Reads with a minimum pairwise identity of 97% were clustered into operational taxonomic units (OTUs) using QIIME’s open-reference OTU-picking workflow, which was based on UCLUST (69) software. The Greengenes bacterial 16S rRNA database (13_8 release) was used for OTU picking (70). The most abundant sequence was chosen to represent each OTU. Taxonomy was assigned to each OTU using QIIME-based wrapper of the Ribosomal Database Project (RDP) classifier (71) against a representative subset of the Greengenes 16S rRNA database 13_8 release, using a 0.50 confidence threshold for taxonomic assignment. Bacterial 16S rRNA gene sequences were aligned using PyNAST (72) against a template alignment of the Greengenes core set filtered at 97% similarity. During the process, chimeras were identified and removed using the ChimeraSlayer (73) algorithm, and a phylogenetic tree was built from the filtered alignment using FastTree (74). Any OTU representing less than 0.001% of the total filtered sequences was removed to avoid erroneous reads that could lead to inflated estimates of diversity (75). After these quality-filtering steps, each sample was represented by less than 150 sequences, and the filtered OTU tables were ready for downstream analyses, such as diversity comparisons and biomarker discovery.

Alpha diversity and beta diversity were calculated within QIIME based on weighted UniFrac (76) distance between samples. Principal coordinates were calculated from the UniFrac distance matrices to decrease the dimensionality of the taxonomic data set into three-dimensional (3D) principal coordinate analysis (PCoA) plots, enabling visualization of sample relationships. To determine whether treatments caused differences in phylogenetic or species diversity, analysis of similarity (ANOSIM) (77) and permutational multivariate analysis of variance (PERMANOVA) (78) were used to test significant differences between sample groups based on weighted UniFrac.

Significant taxonomic differences between sample groups were also tested using the linear discriminant analysis (LDA) effect size (LEfSe) (79). LEfSe is an algorithm for high-dimensional biomarker discovery and identification of genomic features (genes, pathways, or taxa) that characterizes the differences between two or more classes/treatments. It first uses the nonparametric factorial Kruskal-Wallis (KW) rank sum test to detect taxa with significant differential abundances with respect to the class of interest (one-against-all strategy). Then LEfSe uses linear discriminant analysis to estimate the effect size of each differentially abundant feature.

### (vi) Fecal SCFAs.

Short-chain fatty acids in the stool samples were analyzed using gas chromatography (GC) according to the method described in a previous study (80) with minor modifications. The GC system consisted of an Agilent 6890 gas chromatograph (Agilent Technologies, Palo Alto, CA), equipped with an automatic sampler (MPS) (Gerstel, Mülheim, Germany) and a flame ionization detector (FID). A high-polarity, polyethylene glycol (PFG), fused silica capillary column DB-WAXETR (30 m, 0.25-mm inner diameter [i.d.], 0.25-mm film thickness) (Agilent Technologies, Palo Alto, CA) was used for separation. Prior to sample analysis, a standard solution containing a mixture of standards (30 mM final concentration of acetic acid, propionic acid, isobutyric acid, butyric acid, isovaleric acid, valeric acid, and caproic acid) in ethyl acetate containing 1 mM heptanoic acid as internal standard (IS) was diluted to obtain a calibration curve ranging from 3 to 3,000 μM. Standard curves were constructed by plotting the concentration of each individual SCFA versus the ratio of SCFA peak area/IS peak area. Each point of the standard curves corresponds to the mean value from three independent injections. Three independent replicate extractions were performed per sample.

### Statistical analysis.

Intention-to-treat (ITT) analyses were applied to treatment effect analyses (81). All data were first tested for normality using the Anderson-Darling test, and log transformation was applied when necessary. The analysis of treatment effect and period effect was performed using analysis of variance (ANOVA) or the Kruskal-Wallis test where appropriate. Mann-Whitney *U* test was used for gender comparison, and the Spearman rho test was employed for correlation analyses; a *P* value of <0.05 was considered significant. All statistical analyses were performed using Minitab 17.0 software (Minitab Inc., State College, PA).

### Data availability.

Sequence data were deposited in NCBI SRA under BioProject PRJNA739252. Metadata and code used for microbiome analyses is available at https://github.com/LauRolon/BB12microbiome.
