# Supplementary material for: Matrix Effects on the Delivery Efficacy of Bifidobacterium animalis subsp. lactis BB-12 on Fecal Microbiota, Gut Transit Time, and Short-Chain Fatty Acids in Healthy Young Adults
Source: mSphere. 2021 Jul 7;6(4):e00084-21. doi: 10.1128/mSphere.00084-21 (PMC8386398; doi:10.1128/mSphere.00084-21)
Supplement: TABLE S1 [file msphere.00084-21-st001.docx]

|  | **ADONIS** | | **ANOSIM** | |
| --- | --- | --- | --- | --- |
|  | **R^2^** | ***P* value** | **R** | ***P* value** |
| **CRP** | 0.007 | 0.372 | 0.041 | 0.316 |
| **TC:HDL-C** | 0.015 | 0.025 | 0.371 | 0.039 |
| **Glucose** | 0.013 | 0.025 | 0.089 | 0.200 |
| **Insulin** | 0.020 | 0.003 | 0.177 | 0.026 |
| **WC** | 0.011 | 0.076 | 0.118 | 0.051 |
| **TG** | 0.010 | 0.116 | -0.092 | 0.800 |
| **HDL-C** | 0.010 | 0.095 | -0.118 | 0.963 |
| **LDL-C** | 0.016 | 0.006 | 0.015 | 0.414 |
| **DCs** | 0.015 | 0.008 | 0.020 | 0.018 |
| **IFN-γ** | 0.018 | 0.003 | 0.019 | 0.033 |
| **TNF-α** | 0.007 | 0.445 | -0.005 | 0.740 |
| **IL-2** | 0.008 | 0.272 | 0.001 | 0.358 |
